# Plasma lipid profiles and homocysteine levels in anti-N-methyl-D-aspartate receptor encephalitis

**DOI:** 10.3389/fneur.2023.1148450

**Published:** 2023-04-13

**Authors:** Zhi-hao Wang, Shan Qiao, Lei Wang, Kemo Wang, Ranran Zhang, Yang Jin, Huai-kuan Wu, Xuewu Liu

**Affiliations:** ^1^Department of Neurology, Qilu Hospital of Shandong University, Jinan, China; ^2^Department of Neurology, The First Affiliated Hospital of Shandong First Medical University and Shandong Provincial Qianfoshan Hospital, Jinan, China; ^3^Department of Neurology of Stroke Center, The First Affiliated Hospital of Soochow University, Suzhou, China; ^4^Department of Interventional Radiology, The First Affiliated Hospital of Shandong First Medical University and Shandong Provincial Qianfoshan Hospital, Jinan, China; ^5^Institute of Epilepsy, Shandong University, Jinan, China

**Keywords:** anti-N-methyl-D-aspartate receptor encephalitis, lipid profile, homocysteine, ApoB/ApoA-1, prognosis

## Abstract

**Introduction:**

We aimed to investigate whether lipid profiles and homocysteine levels in patients with anti-N-methyl-D-aspartate receptor encephalitis are related to clinical presentation and prognosis, which may contribute to further research on the pathogenesis and treatment of this disease.

**Methods:**

This study included a total of 43 patients with anti-N-methyl-D-aspartate receptor encephalitis and 43 sex–age-matched healthy controls. Baseline demography, clinical data, patient outcomes, and ancillary examination results were recorded. Patients were followed up every 2–3 months during the first year. The modified Rankin Scale score was used to evaluate the therapeutic effect and clinical outcome.

**Results:**

Among the 43 patients included in this study, 55.81% were male, the mean age of onset was 27 years old, and the median modified Rankin Scale score on admission was 3.0. Apolipoprotein A-1 was significantly lower in patients with anti-N-methyl-D-aspartate receptor encephalitis compared with healthy controls (*p* = 0.004). Compared with healthy controls, homocysteine (*p* = 0.002), apolipoprotein B (*p* = 0.004), Lpa (*p* = 0.045), and apolipoprotein B/apolipoprotein A-1 (*p* = 0.001) were significantly increased in patients with anti-N-methyl-D-aspartate receptor encephalitis. According to the modified Rankin Scale scores, 6 months after discharge, 72.09% of patients had a good prognosis and 27.91% had a poor prognosis. In the good prognosis group, age (*p* = 0.031), lipoprotein a (*p* = 0.023), apolipoprotein A-1 (*p* = 0.027) at baseline, and the modified Rankin Scale score on admission (*p* = 0.019) were significantly higher than those in the poor prognosis group.

**Conclusion:**

This study suggests the possibility that serum lipid profile and homocysteine play an important role in the pathogenesis of anti-N-methyl-D-aspartate receptor encephalitis, providing support for lipid-lowering treatment of anti-N-methyl-D-aspartate receptor encephalitis patients.

## Introduction

Anti-N-methyl-D-aspartate receptor (anti-NMDAR) encephalitis was first proposed by Dalmau et al. in 2007 and is currently the most common autoimmune encephalitis (1). Clinical manifestations of anti-NMDAR encephalitis include cognitive impairment, behavioral disorder, autonomic dysfunction, epileptic seizures, and changes of consciousness (2–4). The pathogenesis of anti-NMDAR encephalitis remains to be clarified, and potential biomarkers indicating disease progression need to be explored.

It has been reported that high-density lipoprotein (HDL-C) has immune regulation, anti-oxidant, and anti-inflammatory effects (5–7). Researchers indicate that higher serum high-density lipoprotein cholesterol (HDL-C) is associated with lower levels of blood–brain barrier (BBB) injury in multiple sclerosis (MS) (8, 9). In neuromyelitis optic spectrum disorder (NMOSD), low HDL-C and high triglyceride (TG) levels were positively associated with poor recovery and relapse (10, 11).

Apolipoprotein A1 (ApoA-1) is an important component of HDL-C, which has anti-inflammatory effects (12). Studies have found that ApoA-1 is associated with a variety of neurological diseases. ApoA-1 levels were inversely associated with disease severity in MS (13, 14), and lower HDL-C and apoA-I levels were also associated with disease severity in SLE patients (15, 16).

Apolipoprotein B (ApoB) is believed to be atherogenic and has been associated with neurological diseases, such as the future risk of Parkinson's disease (PD) (17). Given the association between lipid profiles and these autoimmune diseases, we hypothesized that lipid profiles might be relevant to the prognosis of patients with NMDAR encephalitis.

Homocysteine (Hcy) is a non-essential sulfur-containing amino acid produced during methionine metabolism and is considered to be a pro-inflammatory and immunomodulatory molecule (18). Hyperhomocysteinemia has been found in a variety of neurological and autoimmune diseases, such as Alzheimer's and Parkinson's diseases (19–21), multiple sclerosis (MS) (22–26), systemic lupus erythematosus (SLE) (27), and rheumatoid arthritis (RA) (28–30). The excitotoxicity and pro-inflammatory effects of Hcy may be induced by the overactivation of NMDA receptors (which exist in neurons, neutrophils, and macrophages) to destroy the blood–brain barrier (BBB) (31), induce excitotoxicity of neurons (32–36), and activate the pro-inflammatory cascade (37).

We aimed to investigate whether lipid profiles and homocysteine levels in patients with anti-NMDAR encephalitis are related to clinical presentation and prognosis, which may contribute to further research on the pathogenesis and treatment of this disease.

## Materials and methods

### Study design and patient selection

This retrospective study was conducted at Qilu Hospital of Shandong University in China from January 2016 to December 2020 and included 43 patients diagnosed with anti-NMDAR encephalitis according to published diagnostic criteria (38). The inclusion criteria were as follows: (1) acute or subacute seizures with one or more clinical features, including seizures, memory deficits, mental and behavioral disorders, and speech disorders related to the limbic system, (2) positive serum and/or cerebrospinal fluid (CSF) for neurosurface antibodies, and (3) reasonable exclusion of other diseases. The exclusion criteria were as follows: (1) patients who had received corticosteroids or lipid-lowering drugs prior to the study; (2) patients having clinical symptoms that affect lipid profiles, such as diabetes, hypertension, hypothyroidism, severe infections, abnormal liver function, abnormal kidney function, or Cushing's syndrome; and (3) patients with incomplete clinical data. In addition, 43 sex- and age-matched healthy individuals were included as a control group.

The modified Rankin Scale (mRS) score was used to evaluate the therapeutic effect and clinical outcome (39). The mRS scores were defined as follows: 0 = asymptomatic; 1 = no significant disability in performing all daily activities despite some symptoms; 2 = having a minor disability in handling personal matters without assistance, but being unable to carry out all previous activities; 3 = moderately disabled, requiring some assistance, but able to walk independently; 4 = moderately severe disability, i.e., inability to take care of physical needs without assistance and inability to walk unassisted; 5 = severe disability requiring constant care and nursing, as well as bedridden and incontinence; and 6 = death.

Patients were followed up every 2–3 months during the first year after discharge; from the second year onward, follow-up was conducted every 4–6 months. Patients were followed up for at least 1 year. Follow-up data were carefully retrieved from hospital records or through interviews with patients and their families (directly or *via* phone and WeChat). After 6 months of discharge, patients with an mRS score of ≤ 2 were defined as the good prognosis group, and patients with an mRS score of >2 were defined as the poor prognosis group.

### Standard protocol approvals, registrations, and patient consents

The study was approved by the Ethics Committee of Shandong University Qilu Hospital (Approval number: KYLL202008-044) and was conducted in accordance with the Declaration of Helsinki. Written informed consent was given to all study participants or their legal guardians.

### Data collection and data criteria

Baseline demography (sex, age, and past medical history), clinical data, patient outcomes, and ancillary examination results were recorded. Autoantibodies to NMDAR were evaluated in 43 patients by indirect immunofluorescence. The initial dilution titers of serum and cerebrospinal fluid were 1:10 and 1:1, respectively. Blood samples were taken from all patients (fasting) on the morning of the second day after the initial admission. Serum lipid profiling and homocysteine levels were measured in our hospital using a chemiluminescent analyzer (Cobas E601, Shanghai, China). The lipid profile included total cholesterol (TC), TG, low-density lipoprotein cholesterol (LDL-C), HDL-C, apolipoprotein A-I (apoA-I), apolipoprotein B, and apolipoprotein A-I/apoB.

### Statistical analysis

SPSS IBM 25.0 software was used for statistical analysis. The Shapiro–Wilk test was used to determine the normality of the values, and the normally distributed continuous variables were expressed as mean and standard deviation. Non-normal data were expressed as median and interquartile spacing (IQR). Class variables were expressed as ratios. Data were analyzed by the chi-square test or Fisher's exact test. Student *t*-test was used for normally distributed continuous variables, and the Mann–Whitney U-test was used for non-normal continuous variables. Bilateral values with a *p-*value of < 0.05 were considered to be statistically significant.

## Results

### Baseline demographics and clinical features of patients with anti-NMDAR encephalitis

**Figure 1:**
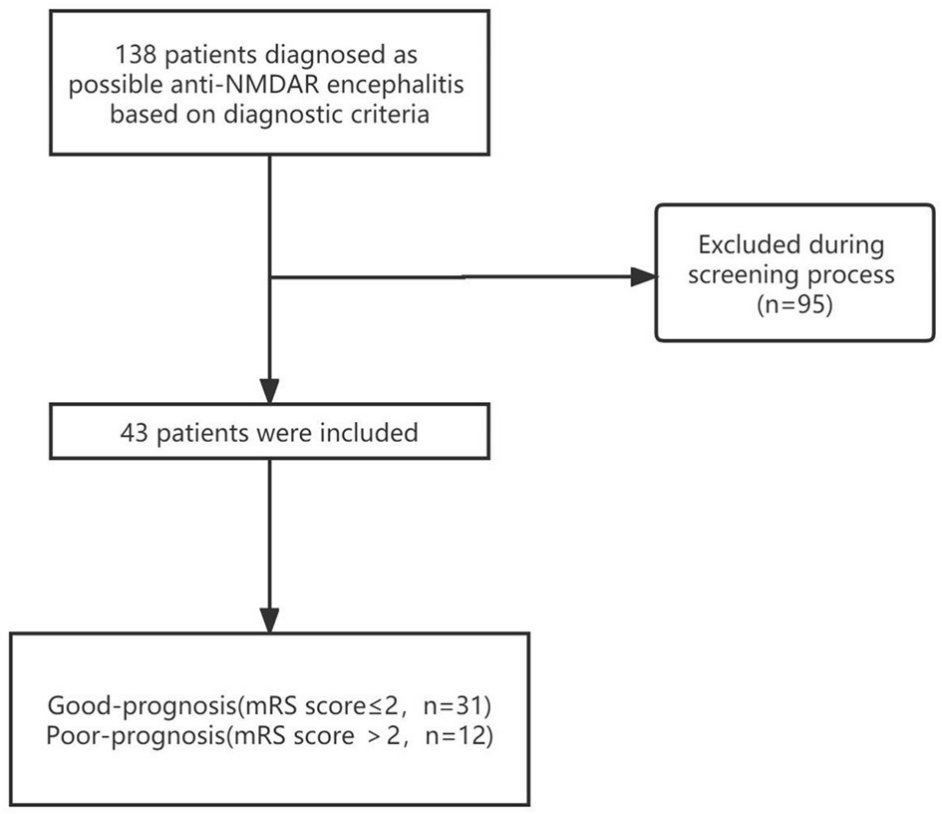
The flowchart of patient recruitment.

From January 2016 to December 2020, clinical data were collected from 138 patients, of whom 95 were excluded because they did not meet the inclusion criteria, had incomplete clinical data, or were lost to follow-up. A total of 43 patients with anti-NMDAR encephalitis and 43 sex–age-matched healthy controls (HCs) were enrolled. Figure 1 depicts the flowchart of patient recruitment. Among the 43 patients included in this study, 55.81% (24/43) were men, the mean age of onset was 27 years (IQR, 18–41), and the median mRS score on admission was 3.0 (3.0, 4.0). The main symptoms of the patients on admission were seizure (23/43), abnormal mental behavior (17/43), memory deficit (7/43), movement disorders (6/43), and consciousness disorders (3/43). In total, two patients were diagnosed with ovarian teratoma after admission and underwent tumor resection during hospitalization. According to the mRS scores, 6 months after discharge, 72.09% (31/43) of patients had a good prognosis and 27.91% (12/43) had a poor prognosis (Table 1). No deaths were recorded during the hospitalization. Overall, one male patient died of severe pneumonia within 6 months of discharge.

**Table 1:** Demographic features of patients with anti-NMDAR encephalitis and healthy controls.

	**Age-and sex-matched CTLs (*n* = 43)**	**Anti-NMDAR encephalitis patients (*n* = 43)**	***p*-value**
Sex(male:female), n	24:19	24:19	–
Age at onset, years	27(18,41)	27(18,41)	–
mRS Scores at admission	–	3.0(3.0,4.0)	
Clinical symptom (n,%)			
Seizures	–	23	–
Memory disorder	–	7	–
Mental behavior disorder	–	17	–
Disturbance of consciousness	–	3	–
Movement disorder	–	6	–
Complicated with tumors	–	2	–
mRS Scores at 6 months after discharge			
≤ 2	–	31	–
>2	–	12	–
TG, mmol/L	1.02 (0.73, 1.52)	1.06 (0.74, 1.23)	0.873
TC, mmol/L	3.91 ± 0.11	4.01 ± 0.12	0.527
HDL-C, mmol/L	1.17 ± 0.04	1.08 ± 0.03	0.11
LDL-C, mmol/L	2.23 ± 0.09	2.25 (1.90, 2.84)	0.56
ApoB/ApoA-1 ratio	0.57 (0.48, 0.77)	0.79 (0.56, 0.96)	0.001
LPa, mmol/L	17.70 (8.3,24.7)	25.6 (9.2, 61.2)	0.045
Hcy, umol/L	11.64 ± 0.35	15.2 (10.8, 19.7)	0.002
ApoA-1, gmol/L	1.26 ± 0.04	1.12 ± 0.03	0.004
ApoB, gmol/L	0.77 ± 0.03	0.91 ± 0.04	0.004

### Comparison of anti-NMDAR encephalitis patients and HCs

As shown in Table 1, ApoA-1 was significantly lower in patients with anti-NMDAR encephalitis compared with healthy controls (*p* = 0.004). Compared with the HCs, Hcy (*p* = 0.002), ApoB (*p* = 0.004), Lpa (*p* = 0.045), and ApoB/ApoA-1(*p* = 0.001) were significantly increased in patients with anti-NMDAR encephalitis. In addition, TG, TC, HDL-C, and LDL-C showed no statistical difference (*p* = 0.873, 0.527, 0.11, 0.56, respectively).

### Subgroup comparison of anti-NMDAR encephalitis patients based on mRS Scores at follow-up after 6 months

According to the mRS scores after 6 months of follow-up, the patients were divided into two groups with mRS scores of ≤ 2 (good prognosis) and mRS scores of >2 (poor prognosis), as shown in Table 2, using the clinical data collected at the baseline for analysis. In the good prognosis group, age (27.34 ± 2.26 vs. 43.58 ± 6.41, *p* = 0.031), lipoprotein a [19.3 (6.5, 41.9) vs. 57.75 ± 11.00, *p* = 0.023], mRS score on admission [3.0 (3.0, 4.0) vs. 4.0 (3.0, 5.0), *p* = 0.019], and ApoA-1 were significantly higher than that in the poor prognosis group [1.15 ± 0.03 vs. 0.99 (0.88, 1.04), *p* = 0.027]. The main symptoms of the good prognosis group on admission were seizure (16/31), abnormal mental behavior (13/31), memory loss (6/31), movement disorders (3/31), and consciousness disorders (3/31), and there was no statistical difference between the good prognosis group and the poor prognosis group. Both patients with ovarian teratoma had a good prognosis.

**Table 2:** Subgroup comparison of anti-NMDAR encephalitis patients based on mRS Scores at follow-up after 6 months.

	**mRS ≤ 2**	**mRS>2**	***p*-value**
Sex (male:female), *n*	17:14	7:5	0.836
Age at onset, years	27.23 ± 2.26	43.58 ± 6.41	0.031
TG, mmol/L	1.06 (0.74,1.21)	1.20 ± 0.15	0.766
TC, mmol/L	4.01 ± 0.15	4.03 ± 0.22	0.913
HDL-C, mmol/L	1.10 ± 0.04	1.03 ± 0.06	0.359
LDL-C, mmol/L	2.25(1.94,2.76)	2.43 ± 0.26	0.850
ApoB/ApoA-1 ratio	0.77(0.53,0.94)	0.89(0.73,1.20)	0.07
LPa, mmol/L	19.3(6.5,41.9)	57.75 ± 11.00	0.023
Hcy, umol/L	15.9(11.3,19.7)	15.73 ± 2.30	0.409
ApoA-1, gmol/L	1.15 ± 0.03	0.99(0.88,1.04)	0.027
ApoB, gmol/L	0.88 ± 0.04	0.98 ± 0.08	0.223
mRS scores at admission	3.0 (3.0,4.0)	4.0 (3.0,5.0)	0.019
Seizures	16	7	0.745
Memory disorder	6	1	0.652
Mental Behavior Disorder	13	4	0.735
Disturbance of consciousness	3	0	0.548
Movement Disorder	3	3	0.325
Complicated with tumors	2	0	1.0

## Discussion

In this regression study, the lipid profile and homocysteine of patients with anti-NMDAR encephalitis were significantly abnormal compared with the HCs. At the baseline, there were statistical differences in ApoA-1, ApoB, ApoB/ApoA-1, LPa, and Hcy between the anti-NMDAR encephalitis patients and the HCs. At the follow-up stage, compared with the poor prognosis group, there were significant differences in age, LPa, mRS score, and ApoA-1 in the good prognosis group.

HDL-C has anti-inflammatory and immune function regulation (5–7). In our study, HDL-C levels did not show statistical significance between the groups. However, in the same type of study on anti-NmdAR encephalitis, it was found that the level of HDL-C in patients was significantly reduced and was associated with poor prognosis and recurrence of patients (40, 41). This may be because our study was small and needed to be larger to show more accurate results.

ApoA-1, as the main component of HDL-C, is involved in a variety of functions, including anti-oxidative stress (42) and inflammatory response (43). ApoA-1 can inhibit endothelial cell apoptosis, pro-oxidation, and pro-inflammatory processes and induce vascular dilation, inhibit platelet activation, and promote innate immunity (44). Although HDL-C did not show statistical significance in this study, ApoA-1 showed low levels in both patients with anti-NMDAR encephalitis and in the group with a poor prognosis. Consistent with our conclusion, low ApoA-1 levels have been found in all the existing studies on anti-NMDAR encephalitis.

In contrast to the anti-inflammatory effects of ApoA-1, ApoB promotes inflammatory progression. Thus, an elevated ApoB/ApoA-I ratio may indicate a pro-inflammatory effect over an anti-inflammatory effect of lipoprotein, indicating the progression of inflammation and severity of disease (45). Our study found that serum ApoB levels and the ApoB/ApoA-I ratio were increased in patients with anti-NmdAR compared with the HCs. In addition, ApoB and ApoB/ApoA-1 in the good and poor prognosis groups showed a higher trend than that in the good prognosis group, although there was no statistical significance. A regression study showed that the level of ApoB/ApoA-1 in patients was significantly higher than that in healthy people but was not related to the outcome of the disease (46). However, another study suggested that ApoB/ApoA-1 was associated with adverse outcomes in patients (41). Given our small sample size, it is possible that a larger study could give a clearer trend.

There is evidence that lipoprotein a (LPa) plays a role in promoting the progression of inflammation in most cases (47). High levels of LPa have been found in both rheumatoid arthritis and systemic lupus erythematosus (48–51). In this study, the level of LPa in anti-NmdAR patients was significantly higher than that of the HCs, and the level of LPa in the poor prognosis group was significantly higher than that in the good prognosis group. This may be related to its pro-inflammatory effect.

Hyperhomocysteinemia has been found in a variety of neurological and autoimmune diseases (19–30). A large number of studies have shown that excessive Hcy can cause neurotoxicity through its dual effect on NMDAR (42), and Hcy can also induce neuronal excitatory toxicity through overstimulation of NMDAR (32–36). In addition, hyperhomocysteinemia may also promote an inflammatory cascade (37). In this study, Hcy levels were significantly increased in patients with anti-NMDAR encephalitis compared with the levels in the HCs, and there was no statistical difference in Hcy levels between the good prognosis group and the poor prognosis group. Studies have shown that the Hcy level of patients with anti-NMDAR encephalitis is significantly higher than that of healthy groups, and that of male patients is higher than that of female patients (52).

There are several limitations to this study that need to be addressed. First, this was a single-center, small sample size, and retrospective study, so more centers and larger sample sizes are needed to further explore the relationship between lipid profiles and homocysteine and anti-NMDAR encephalitis. Second, the potential impact of treatment remains a confounding variable. Finally, due to the limited sample size, anti-NMDAR encephalitis patients were only divided into good prognosis and poor prognosis groups for subgroup analysis. A more detailed subgroup analysis may be more helpful in studying the role of lipid profiles and homocysteine in the disease.

## Conclusion

In conclusion, our results showed that ApoB, ApoB/ApoA-1, Hcy, and LPa levels significantly increased and ApoA-1 levels decreased in patients with anti-NMDAR encephalitis at the early stage of the disease. Follow-up results of 6 months after discharge showed that mRS scores and the LPa level in the poor prognosis group were significantly higher than those in the good prognosis group at the age of onset and the early stage of onset, and ApoA-1 levels were significantly lower at the early stage of onset than those in the good prognosis group. In addition, there are several threshold variables in this study, and larger studies may be needed to further clarify the actual situation of such variables. With our research, it is still difficult to determine the role of lipids and homocysteine in the pathogenesis of anti-NMDAR encephalitis, but in view of their differences between patients with anti-NMDAR encephalitis and healthy people, as well as between different prognoses, we believe that lipids and homocysteine can be used to detect or predict patients' condition. Larger samples are needed to further elucidate the relationship between serum lipid profiles and anti-NMDAR encephalitis.

## Data availability statement

The raw data supporting the conclusions of this article will be made available by the authors, without undue reservation.

## Ethics statement

The studies involving human participants were reviewed and approved by the Ethics Committee of Shandong University Qilu Hospital (Approval number: KYLL202008-044) and was conducted in accordance with the Declaration of Helsinki. Written informed consent to participate in this study was provided by the participants' legal guardian/next of kin.

## Author contributions

Z-hW and SQ conceptualized and designed the study, collected and organized the data, drafted the initial manuscript, reviewed, and revised the manuscript. LW, KW, RZ, YJ, and Z-hW assisted in collecting data and provided important suggestions for the design of research scheme and the writing of manuscript. XL conceptualized and designed the study, coordinated and supervised data collection, and critically reviewed the manuscript for important intellectual content. All authors approved the final manuscript as submitted and agree to be accountable for all aspects of the work.
